# Island Cotton *Gbve1* Gene Encoding A Receptor-Like Protein Confers Resistance to Both Defoliating and Non-Defoliating Isolates of *Verticillium dahliae*

**DOI:** 10.1371/journal.pone.0051091

**Published:** 2012-12-10

**Authors:** Baolong Zhang, Yuwen Yang, Tianzi Chen, Wengui Yu, Tingli Liu, Hongjuan Li, Xiaohui Fan, Yongzhe Ren, Danyu Shen, Li Liu, Daolong Dou, Youhong Chang

**Affiliations:** 1 Provincial Key Laboratory of Agrobiology, Jiangsu Academy of Agricultural Sciences, Nanjing, China; 2 College of Plant Protection, Nanjing Agricultural University, Nanjing, China; East Carolina University, United States of America

## Abstract

Verticillium wilt caused by soilborne fungus *Verticillium dahliae* could significantly reduce cotton yield. Here, we cloned a tomato *Ve* homologous gene, *Gbve1*, from an island cotton cultivar that is resistant to Verticillium wilt. We found that the *Gbve1* gene was induced by *V. dahliae* and by phytohormones salicylic acid, jasmonic acid, and ethylene, but not by abscisic acid. The induction of *Gbve1* in resistant cotton was quicker and stronger than in *Verticillium*-susceptible upland cotton following *V. dahliae* inoculation. *Gbve1* promoter-driving GUS activity was found exclusively in the vascular bundles of roots and stems of transgenic *Arabidopsis*. Virus-induced silencing of endogenous genes in resistant cotton via targeting a fragment of the *Gbve1* gene compromised cotton resistance to *V. dahliae*. Furthermore, we transformed the *Gbve1* gene into *Arabidopsis* and upland cotton through *Agrobacterium*-mediated transformation. Overexpression of the *Gbve1* gene endowed transgenic *Arabidopsis* and upland cotton with resistance to high aggressive defoliating and non-defoliating isolates of *V. dahliae*. And HR-mimic cell death was observed in the transgenic *Arabidopsis*. Our results demonstrate that the *Gbve1* gene is responsible for resistance to *V. dahliae* in island cotton and can be used for breeding cotton varieties that are resistant to Verticillium wilt.

## Introduction

*Verticillium dahliae* Kleb is a soil-borne fungus that penetrates the vascular system of the plant and causes vascular wilt disease. This pathogenic fungus survives in the soil for long periods as microsclerotia, tiny structures produced in the plant tissue. It infects hundreds of economically important crops and trees, but unfortunately, no currently available fungicides are able to effectively control the disease [1]. Verticillium wilt in cotton has been reported in most cotton-growing areas, and it has become the most important disease of cotton in the world [2]. For example, 5–6.6 million acres, approximately half of the cotton cultivating area in China, were subjected to this disease in 2009 and 2010 (National Cotton Council of America– Disease Database). So far, the most effective strategy to combat this stubborn disease is to develop tolerant cotton cultivars by incorporating genes from resistant germplasm. However, higher level of Verticillium wilt resistance exists in island cotton but not upland cotton [3].

Isolates of *V. dahliae* have been characterized based on virulence capacity and the phenotypic symptoms occurring in disease development. *V. dahliae* isolates from cotton are considered either defoliating isolates that cause severe leaf wilting and shedding in infected plants or non-defoliating isolates that induce leaf-wilt symptoms, but cause only limited shedding of the leaves during disease progression [4]. In general, defoliating isolates may possess a stronger pathogenic capacity. BP2 and V991 are two widely spread *V. dahliae* isolates found in China and represent medium aggressive non-defoliating and highly aggressive defoliating isolates, respectively [5], [6].

Efforts made to utilize genetic engineering to obtain transgenic cotton resistant to *V. dahliae* have had varying results. Genes used for these studies have included *GbVe* [7], *Arabidopsis NPR1* [8], [9], anti-apoptotic gene *p35* [10], HR-induced *Hpa1Xoo* [11] and some antifungal genes, including chitinase [12], D4E1 [13], lipid transfer proteins [14] and gastrodianin [15]. The resistance levels of transgenic seedlings range from the complete inhibition of *Verticillium* growth in engineered plant extracts [12], [13], to enhanced disease resistance in field trials [7], [10], [11], [15] or increased resistance to only weak pathogen varieties [8]. For example, the number of germinating *V. dahliae* conidia was significantly reduced in plant extracts from transgenic cotton overexpressing a synthetic antimicrobial peptide, D4E1 [13]. *AtNPR1*-transgenic cotton exhibited significant resistance against non-defoliating isolates but remained susceptible to the defoliating isolates of *V. dahliae* [8].

Effective control of Verticillium wilt has been reported in specific crops exhibiting race-specific resistance. The *Ve* locus of tomato is the only cloned locus that is responsible for resistance against race 1 strains of *V. dahliae* and *V. albo-atrum*. This locus includes two proteins named Ve1 and Ve2, leucine-rich repeat class of receptor-like proteins (eLRR-RLPs) [16]. Both genes confer resistance when expressed in the close relative potato, while only Ve1 provides resistance in both tomato [17] and in transgenic *Arabidopsis* [18]. The eLRR-RLPs class proteins differ from receptor-like kinases in that they lack a cytoplasmic kinase domain and carry only a short cytoplasmic tail that lacks obvious signaling motifs [19], [20]. This class of race-specific R proteins also includes Cf proteins conveying *Cladosporium fulvum* resistance in tomato [21] and apple HcrVf proteins that convey resistance against *Venturia inaequalis* [22]. *Ve1*-mediated resistance signaling is activated by the effector Ave1. Ave1 is encoded by race 1 strains of *V. dahliae* and is homologous to a widespread family of plant natriuretic peptides [23]. Genetic analysis has shown that this signaling pathway in tomato requires *EDS1* (Enhanced Disease Susceptibility 1), *NDR1* (Non-race-specific Disease Resistance 1), *BAK1* (BRI1-Associated Kinase 1), *MEK2* (*MKK2*, MAP kinase kinase 2), and only partially overlaps with signaling mediated by Cf proteins [17]. *Ve*-mediated resistance involves several short- and long-term defensive mechanisms, including the onset of hydrogen peroxide (H_2_O_2_) production, activities of peroxidase and PAL (Phenylalanine ammonia lyase), and the synthesis of lignins [24].

Four species of the *Gossypium* genus are cultivated in agriculture, including two allotetraploids (*G. hirsutum* and *G. barbadense*) and two diploids (*G. herbaceum* and *G. arboreum*). Island cotton possess higher resistance toward Verticillium wilt than upland cotton [3], which produces more than 95% of the annual cotton crop worldwide [25]. However, cross-breeding between island and upland cotton is a challenging work. Silencing *NDR1*, *MKK2*, or *Ve1*-like genes in *Gossypium hirsutum* compromises resistance to Verticillium wilt [26], indicating that *Ve1*-mediated resistance signaling is conserved between tomato and cotton, and required for resistance to *V. dahliae* infection. These findings suggest that resistant seedlings of upland cotton may be obtained by expressing a *Ve*-like gene from *Gossypium barbadense*, which has been confirmed by a recent report in which a *GbVe* was cloned and enhanced resistance to *Verticillium* wilt in transgenic *Arabidopsis* plantlets [7].

Here, a *Ve* homologous gene, *Gbve1*, which conferred resistance to both defoliating and non-defoliating isolates of *V. dahliae* in cotton, was verified. qRT-PCR revealed that the *Gbve1* gene was induced by *V. dahliae*. GUS stain analysis indicated that the *Gbve1* gene driven by its promoter was specifically expressed in the vascular bundles of roots and stems. Silencing of *Gbve1* in Verticillium wilt-resistant cotton H7124 compromised cotton resistance to *V. dahliae*. Furthermore, transgenic *Arabidopsis* and cotton exhibiting overexpression of the *Gbve1* gene displayed strong resistance to *V. dahliae*.

## Materials and Methods

### Plant Materials, *Verticillium dahliae* and Culture Conditions

Verticillium wilt resistant island cotton cv. H7124 (*G. barbadense*) and susceptible upland cotton cv. Yumian 1 (*G. hirsutum.*) were cultured at the conditions of emperatures ranging from 20°C to 25°C, under a 16/8 h photoperiod and at 80% relative humidity. *A. thaliana* ecotype Columbia-0 were germinated on the half strength MS medium and transferred into pots containing vermiculite soil in the plant incubator with temperature at 25°C day and 20°C night, 60–70% relative humidity, and light intensity of 200 umol/m^−2^/sec^−1^ on a 16 h light/8 h dark cycle.

Highly aggressive defoliating isolate V991 and non-defoliating isolates BP2 of *Verticillium dahlia* were freshly isolated from infected cotton plants and maintained on potato dextrose agar (PDA) at 25°C for 7 d, followed by inoculation into Czapek’s medium in 1 liter of distilled water. These two *V. dahlia* isolates were used to the inoculation of *A. thaliana* and cotton. Before *V. dahliae* inoculation, the conidia were counted and the conidia suspension was adjusted to a needed density with distilled water.

### Gene Cloning, Bioinformatics and Expression Analysis of *Gbve1*

Genomic DNA was extracted from plant tissues by the CTAB method [27]. While total RNA was extracted from plant tissues with an RNAiso Kit (TaKaRa) according to the manufacturer’s protocol, then subjected to RNase-free DNase I (TaKaRa) digestion and purification. An aliquot of 2 µg RNA were used to synthesize the first-strand cDNA using a Primscript RT-PCR kit (TaKaRa) according to the manufacturer’s instructions.

The primers EST- F/EST- R specific to a cotton EST (TC121084) in gene index (http://compbio.dfci.harvard.edu/tgi/plant.html), which was highly homologous to the tomato *Ve1* gene, were used to amplify the genomic DNA of H7124. The amplified sequence was subsequently used as probe to screen a *G. hirsutum* cv. Maxxa BAC library [28]. A clone containing a full open reading frame homologous to tomato *ve1* (named *Ghve1*) was further analyzed in this study. The specific primers Vdr2-cF/Vdr2-cR were designed according the *Ghve1* gene and used to amplify its homology in *G. barbadense* with genomic DNA and cDNA of H7124 and Yumian1, respectively. The upstream sequence of *Gbve1* gene was obtained by genome walking according to the GenomeWalker™ (Clontech) instruction with two pairs of primers AP1/GSP1 and AP2/GSP2. All the PCR products were cloned into pGEM-T Eeasy vector (Promega), transformed into *E. coli* DH5α and sequenced.

Nucleotide sequences and deduced amino acid sequences of *Gbve1* gene were compared using DNAman software (Lynnon Biosoft). The putative motif and domains were identified with SMART, TMHMM and SignalP 3.0 tools and software packages. The flanking sequence of *Gbve1* gene was analyzed with the PLACE database at the Advanced Biosciences Computing Center [29]. Multiple sequence alignments were made using Muscle 3.8 [30] under the default settings. The phylogenetic tree of *Ve* like genes was constructed using MEGA 4.1 [31] with Neighbor-Joining method. The bootstrap analyses were performed with 1000 replications.

At 2-leaf-stage, cotton plants were challenged by *V. dahliae* by soil drenching [32], pouring 10 ml conidial suspension (1×10^7^ conidia/ml) in the soil beside the plants in 750 ml- pots. For hormone treatment, seedlings at 4-leaf-stage were sprayed with 10 mM salicylic acid (SA), ethylene released from 5 mM ethephon(ETH), 100 µM abscisic acid (ABA) and 100 µM methyl jasmonate (MeJA), followed by covering with plastic bags to keep 100% humidity. Stem tissues from *V. dahliae* infected plants and leaf tissues from hormone treated plants were harvested at an appropriate time for RNA extraction.

Quantitative reverse transcription-polymerase chain reaction (qRT-PCR) for *Gbve1* gene was conducted using a SYBR Premix ExTaqTM II Kit (TaKaRa) with cotton poly-ubiquitin 14 (UBQ14) gene [33] as the internal standard. The PCR program contained an initial denaturation step of 1 min at 95°C, followed by denaturation for 15 s at 95°C, annealing for 20 s at 60°C, and extension for 20 s at 72°C for 40 cycles. The real-time PCR thermal cycler qTOWER 2.0/2.2 (Analytik jena, Germany) was used to obtain relative expression levels of each sample. All qRT-PCR expression assays were independently performed and analyzed three times under identical conditions. All the primers used in this paper were list in Table S2.

### VIGS in Cotton

The vectors for virus-induced gene silencing (VIGS) in cotton were obtained from Dr. Xueping Zhou in Zhejiang University. Briefly, the constructs containing *Gbve1* gene were generated from the *geminivirus* cotton leaf crumple virus (CLCrV) vectors [34] and modified for agrobacterium mediated infection method. *Agrobacterium* cultures containing CLCrV-A-*Gbve1* and CLCrV-B were mixed at a 1∶1 ratio and infiltrated into two fully expanded cotyledons using a needle-less syringe. After agroinfection, the cotton plantlets were kept in incubator with temperature 25°C and relative humidity 80%. The *ChlI* gene, which encodes one unit of the chloroplast enzyme magnesium chelatase required for chlorophyll biosynthesis, was used as control, since a visible phenotype of yellow-colored leaves was observed when the *ChlI* gene of cotton was targeted by VIGS [34]. 15–20 d post agroinfection, all the VIGS treated plantlets were checked the replication of CLCrV by PCR with specific primers Clcrv-F and Clcrv-R using genomic DNA as template, and positive plantlets were then subjected to *V. dahliae* inoculation by soil drenching with 30 ml conidial suspension (1×10^6^ conidia/ml) for each pot (250 ml). Foliar damage was evaluated by rating the symptom on the cotyledon and leaf of inoculated plant according to the following disease grades: 0 =  health plant, 1 =  yellowing or necrosis of 1–2 cotyledons, 2 =  yellowing or necrosis of 1true leaf, 3 =  more than 2 wilted or necrotic leaves, 4 =  no leaf left or dead plant. The disease index was calculated according to the following formula: disease index = [(∑disease grades × number of infected)/(total checked plants×4)]×100. VIGS experiments were repeated three times with more than 15 plants each time.

### Generation and Analysis of Transgenic *Arabidopsis*

The full open reading frame of *Gbve1* was amplified with primer pairs Gbve1-*Sma*IF/Gbve1-*Sac*IR, and cloned into the pCAMBIA2301 vector. The promoter fragment of *Gbve1* gene was amplified with primers Pro-F/Pro-R, and introduced into the pBI101.1 binary vector to drive the *GUS* gene [35]. Both constructs were confirmed by sequencing before transformation into *Agrobacterium tumefaciens* strain LBA4404. The *Arabidopsis* transformation was carried out with floral dip [36], and transgenic plants were selected on MS medium containing 50 mg/l kanamycin and 500 mg/l cefotaxine.

To assess the *V. dahliae* resistance of transgenic *Arabidopsis* with *Gbve1* gene, *Arabidopsis* plants in pots (200 ml) were subjected to soil drench inoculation with 10 ml conidial suspension (1×10^6^ conidia/ml). Plants in the control group received same amount of sterile water. The degree of *V. dahliae* infection was divided into five disease grades with disease scores ranging between 0 and 4(0 = healthy plants, no fungal infection, 1 = 25% of the leaves showing yellowing or abnormal yellow spots, 2 = 25 to 50% of the leaves showing yellow spots, 3 = 50 to 75% of the leaves showing brown spots and curled leaf edges with some leaves dropping, 4 = ≥75% of the leaves produce yellow or yellow irregular spots between the main vein of leaves. The disease index was calculated as the method described formula above. At least 16 T3 individual plants per line were subjected to resistant analysis and each experiment was set 3 times.

To investigate the resistance mechanism of *Gbve1* gene in transgenic *Arabidopsis*, *Arabidopsis* leaves were inoculated with the 10 µl spore drop of *V. dahliae* (1×10^7^ conidia/ml) and then incubated at 25°C in a moist incubator for 2 days, the infected leaves were then stained with Lactophenol-Trypan Blue [37] and examined by the Olympus 1X71 invert microscope. Moreover, the expression of four internal defense related genes *PR1*, *PR5*, *EDS1* and *GST1* genes [38] were compared by qRT-PCR between transgenic *Gbve1* gene *Arabidopsis* and the wild type, with *TUB* gene as the internal standard. Histochemical localization of GUS activity in transgenic *Arabidopsis* with *Gbve1* promoter construct was performed according to Jefferson [35].

### Generation and Analysis of Transgenic Cotton

The *Agrobacterium* transformation of embryogenci calli of upland cotton WC line was conducted according to the protocol described by Wu [39]. The regenerated T0 and T1 plants were subjected to southern blot and qRT-PCR test, respectively. For *V. dahliae* inoculation, T1 seedlings were planted in vermiculite soil in paper pots (250 ml). At the 2-leaf-stage, cotton plants were inoculated with *V. dahliae* by soil drenching with 30 ml conidial suspension (1×10^6^ conidia/ml) for each pot (250 ml), followed by keeping in the incubator with 25°C temperature, a 16/8 h photoperiod and 80% relative humidity. Foliar damage and disease index in cotton was evaluated by the method described above.

## Results

### Isolation of a Receptor-like Protein Gene from Cotton

**Figure 1:**
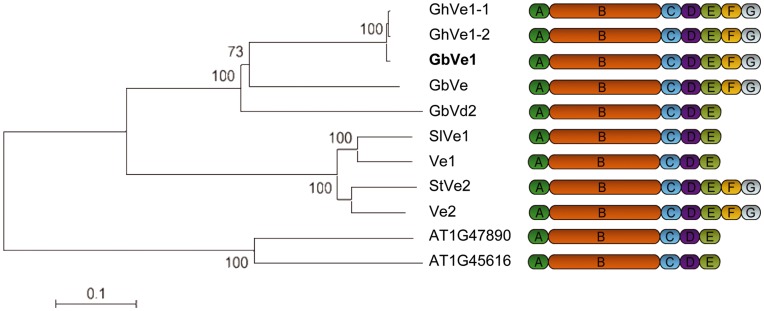
Phylogenetic and structural analysis of Ve-like genes. Domain A is the predicted signal peptide. Domain B that constitutes the main body of the protein is mainly composed of LRRs. The membrane-associated hydrophobic domain D is flanked by a neutral/acidic amino acids rich domain C and by a neutral/basic amino acids rich domain E. Domain F contains a PEST sequence, while domain G contains an endocytosis signal. The detail information about the sequences and domains of GbVe1 protein are shown in Figure S1. Other genes could be found in the GenBank database using following accession numbers: GbVe (EU855795), GbVd2 (GU299534), SlVe1 (AY262016), StVe (AY311527), Ve1 (AF272367), Ve2 (AF365929), and the two *A. thaliana* genes, AT1G47890 and AT1G45616, could be found in www.arabidopsis.org.

To clone the tomato *Ve* homologous gene in cotton, we screened the cotton Bac libraries using a cotton EST that shares 48% similarity in protein with tomato Ve1 protein. A gene was obtained from a total of 40 independent positive clones. Sequence analysis showed that this gene was 76% homologous to the original EST. Then, we amplified the gene allele from the island cotton cultivar H7124, which is resistant to Verticillium wilt, an upland cotton cultivar Yumian1, which is sensitive, and an upland cotton cultivar Changkangmian, which is moderately resistant. These three genes were designated as *Gbve1*, *Ghve1* and *Ghve1-2*, respectively. Comparing the sequence amplified by DNA and cDNA indicated that *Gbve1* has no introns. The *Gbve1* gene was predicted to encode a protein with 1138 amino acids and it shared many similarities with tomato Ve1 and Ve2 proteins, and other Ve-like proteins in cotton [7], [26], *Solanum licopersicoides* [40], and *Solanum torvum* [41]. Each of these proteins contained the predicted signal peptide (domain A), multiple copy LRR domain B, neutral/acidic amino acid-rich domain C, membrane-associated hydrophobic domain D, and a positively charged cytoplasmic domain E. The predicted C-terminus of Gbve1 protein also contained the PEST sequence (domain F) and endocytosis signal (domain G) found in the Ve2 protein (Figure 1; Figure S1). The similarities between both protein sequences and the deduced primary structures of GbVe1 and Ve1/Ve2 proteins indicate that *GbVe1* may play a similar role.

### *Gbve1* Induction by *V. dahlia*, SA, JA, and ET, but not ABA

**Figure 2:**
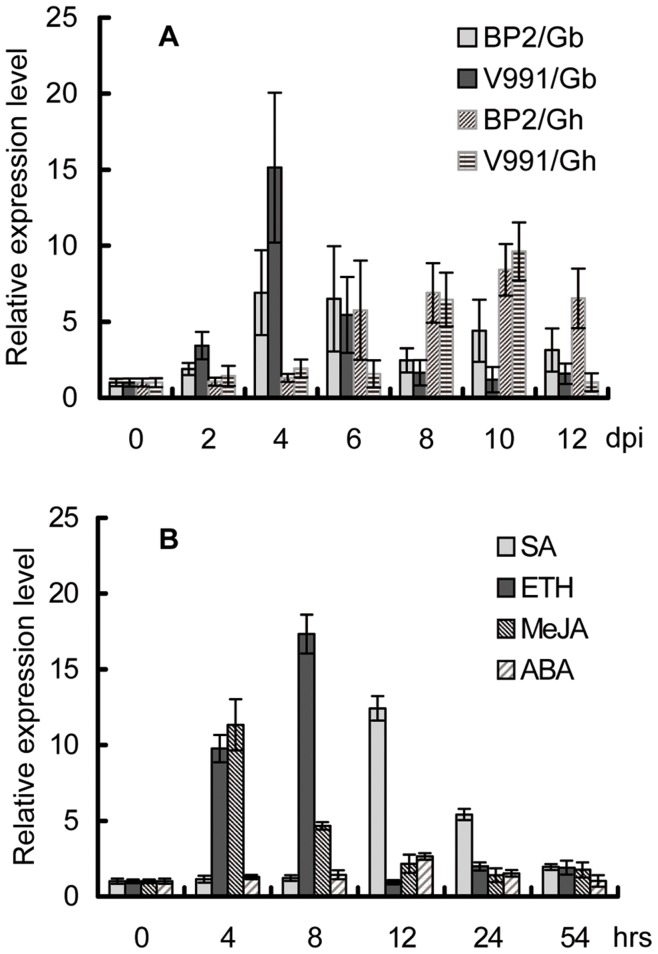
Expression patterns of the *Gbve1* and *Ghve1* genes. (A). Expression patterns of *Gbve1*/*Ghve1* genes in H7124 (Gb) and Yumian 1 (Gh) under infection by *V. dahliae* isolate V991 and BP2. dpi means days post inoculation. (B). Expression patterns of the *Gbve1* gene by different phytohormones.

qRT-PCR was performed to evaluate the relative expression of the *Gbve1* gene in stems of resistant (H7124) and susceptible cotton genotypes (Yumian1) after inoculation with the defoliating and non-defoliating isolates of *V. dahliae*, respectively. Although gene expression in both cultivars was highly induced upon infection with pathogens, both response time and induction level were dramatically different between the incompatible and compatible interactions. Figure 2A shows that the expressional level of the *Gbve1* gene in H7124 reached a peak at 4 dpi (days post inoculation) and then dropped to a similar level to that of untreated tissue at 8 dpi. In contrast, induction patterns in Yumian1 were much slower and weaker and with a peak appearing at 10 dpi. Also, induction levels of the defoliating isolate (V991) were more intense than those of the non-defoliating isolates (BP2) in the resistant cultivar H7124.

The promoter region (1.5 kb upstream) of the *Gbve1* gene was cloned and sequenced. Bioinformatic analysis revealed many regulated elements related to pathogen infection and defense response, including SA-responsive elements, ETH activation sites, elicitor or wounding responsive transcription elements, MYB transcriptional factor recognition, or ABA-responsive sites (Table S1). These results indicate that expression of the *Gbve1* gene may be induced by defense signaling molecules in addition to pathogen infection. Therefore, we examined the *Gbve1* expression profiles upon treatment with SA, ETH, and MeJA. The *Gbve1* transcripts were induced by all the three tested hormones. The highest level of transcript was shown at 4, 8, and 12 h after treatment with MeJA, ETH, and SA, respectively. The induced transcript levels of *Gbve1* were 11.3–16.2-fold higher than those of untreated tissue. In a control of induction by ABA, the maximum amount of *Gbve1* transcripts appeared after 12 h, and transcript levels were only 3-fold higher than the untreated control (Figure 2B).

**Figure 3:**
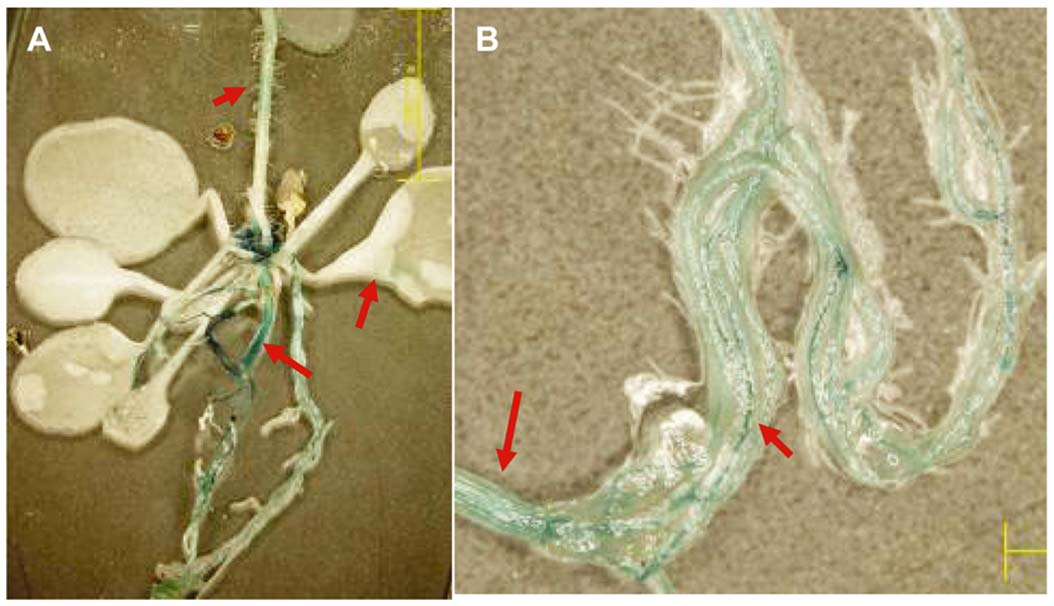
GUS activities in transgenic *Arabidopsis* plants containing the PGbve1:GUS construct. (A). GUS activities in the *Arabidopsis* plantlet. (B). Magnified view of vascular bundles in roots and stems.

Furthermore, we investigated the expressional patterns of the *Gbve1* gene in *Arabidopsis* using transgenic plant lines that were modified to have the promoter region of the *Gbve1* gene fused to the *GUS* gene. The leaves and petioles were faintly blue, while roots and stems always showed high GUS activity (Figure 3A). Microscopic analysis revealed that GUS activity was found exclusively in the vascular bundles of roots and stems (Figure 3B). Thus, this gene may be induced by defense signaling molecules and infection by *V. dahliae* in the vascular regions of roots and stems. The induction was more rapid and dramatic in the resistant cultivar than in the susceptible cultivar.

### *Gbve1*’s Necessity in Verticillium Wilt Resistance in Island Cotton

**Figure 4:**
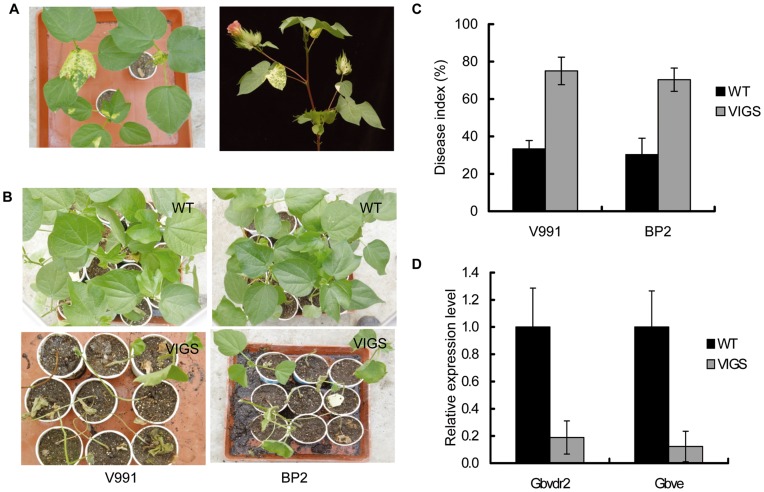
The *Gbve1* gene endowed Verticillium wilt resistance in cotton. (A). The cotton *chlI* gene was used as a positive control with a phenotype of yellow-colored leave after VIGS in cotton (Left: 3 weeks; Right: blossom stage). (B). The phenotypes of H7124 under infection by *V. dahliae* isolates V991 and BP2 after VIGS with CLCrV containing a fragment of *Gbve1* gene. Photos were taken at 42 d after *V. dahliae* inoculation (55 d after VIGS). (C). The disease indices *Gbve1* gene-silence lines. The results were presented as means±SE from three replications with at least 25 plants per replication. (D). Expression levels of the *Gbve1* gene and its homolog (the *Gbve* gene) in the silenced lines.

Since virus-induced gene silencing (VIGS) has been successfully used in the cotton previously [26], [34], [42], we used this approach to further investigate the roles of *Gbve1* in resistance to *Verticillium* wilt. The cotton *chlI* gene was used as a control to monitor the efficiency of VIGS in cotton [34]. Two to three weeks after agroinfiltration with the *chlI* gene, leaves displayed the yellow phenotype (Figure 4A). We evaluated the levels of *V. dahliae* resistance in the *Gbve1*- deficient cotton lines and used the infiltration of CLCrV-A empty vector as a control. In general, more than 80% of the *Gbve1* VIGS plants were severely infected by the two tested isolates of *V. dahliae* (Figure 4B), causing all the leaves to wilt in some cases. The average disease indices of *Gbve1* VIGS plants were 75 and 70 by V991 and BP2, respectively. Average disease indices of the control plants were 33 and 30 (Figure 4C). The silencing efficiencies of the *Gbve1* gene were further confirmed by qRT-PCR analysis using RNA isolated from leaves of VIGS and control plants. Considering that the *GbVe* gene shared many similarities with the *Gbve1* gene [7], we simultaneity measured the expressional levels of the *GbVe* genes. Figure 4D showed that the expression level of both genes dropped approximately 81–89% in VIGS plants compared to the control. As expected, *Gbve1*- silencing plants and plants infected with the empty vector exhibited the similar growth phenotypes as the wilt type (Figure S2). Therefore, VIGS assays confirmed that *Gbve1* and/or *GbVe* genes are important components in island cotton resistance to *V. dahliae* infection.

### *Gbve1* Conferred Verticillium wilt Resistance in Transgenic *Arabidopsis*

**Figure 5:**
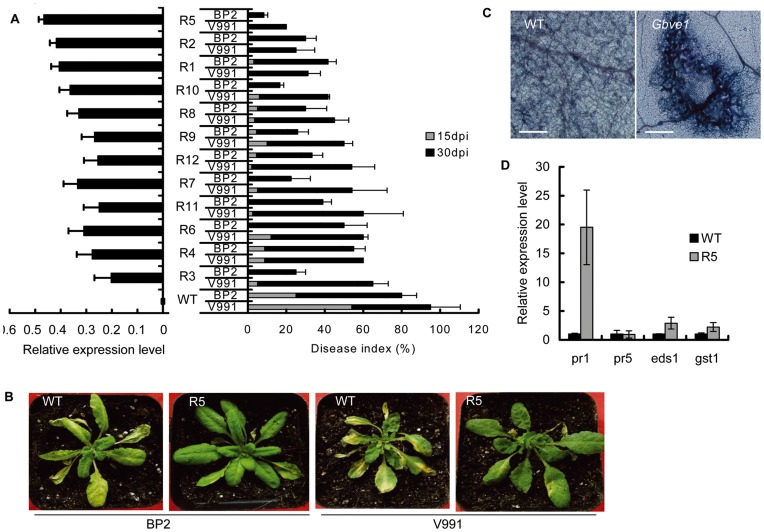
*Gbve1* confers Verticillium wilt resistance in transgenic *Arabidopsis*. (A). The resistant levels of transgenic *Arabidopsis* (left panel) to *V. dahliae* was related to the expression levels of the *Gbve1* gene in the T_3_ independent transgenic lines (right panel). The disease indices were presented means±SE from three replications with at least 16 plants per replication. (B). Phenotype comparison of transgenic line R5 and WT infected with *V. dahliae* isolates V991 and BP2. (C).The *Gbve1* transgenic *Arabidopsis* were resistance to *V. dahliae* through cell death response. Bar = 200 µm. (D). Expressional levels of genes relative to pathogen resistance in transgenic line R5.

To further explore the function of *Gbve1* in *V. dahliae* resistance, the coding sequence of this gene was inserted into the plant expression vector pCAMBIA2301 and transformed into a *V. dahliae*-susceptible *Arabidopsis* genotype Columbia. Seventeen independent transgenic lines were obtained by kanamycin-resistance selection and confirmed by PCR verification. RT-PCR analysis further confirmed that *Gbve1* was successfully expressed in 12 transgenic lines (Figure 5A). Four-week-old seedlings of a T_3_ generation were used to analyze Verticillium wilt resistance by soil drenching methods. At 15 dpi, the disease index of 12 transgenic lines ranged from 0 to 12 due to V991 infection and from 0 to 8.75 because of BP2 infection, with no disease symptoms observed from V991 infection in three lines and no disease symptoms observed from BP2 infection in seven lines. In contrast, a disease index of 54 was scored for V991 infection, and 25 for BP2 infection (Figure 5A). At 30 dpi, the disease index in WT plants increased to 95 and 80 from V991 and BP2 infection, respectively, while the index in transgenic lines varied from 20 to 65 due to V991 infection and 16.7 to 55 because of BP2 infection. Transgenic lines R5 and R2 at 30 dpi showed excellent Verticillium wilt resistance with a disease index of less than 30 under either V991 or BP2 inoculation (Figure 5A, 5B). The resistance levels of transgenic *Arabidopsis* to *V. dahliae* were highly correlated with the expression levels of the *Gbve1* gene in these transgenic lines (Figure 5A).

We observed the cell death development under microscope and found that the *V. dahliae* hypha in wild type leaves were easy to extend to adjacent cells and none cell death was shown at 2 days. In contrast, the *Gbve1* transgenic lines exhibited strong hypersensitive response (HR) cell death, and it restricted the extension of *V. dahliae* hypha (Figure 5C). We also analyzed some genes reported to be involved in pathogen resistance in transgenic *Arabidopsis* and WT plants. Among the four tested genes, PR1 was confirmed to increase about 20-fold in the *Gbve1* transformed plants compared to WT plants. The expression level of *EDS1* and *GST1* in the *Gbve1* overexpressed plants was nearly double that of the WT, whereas no difference in *PR5* was observed between *Gbve1* overexpressed plants and WT plants (Figure 5D).

### *Gbve1* Conferred Verticillium Wilt Resistance in Transgenic Upland Cotton

**Figure 6:**
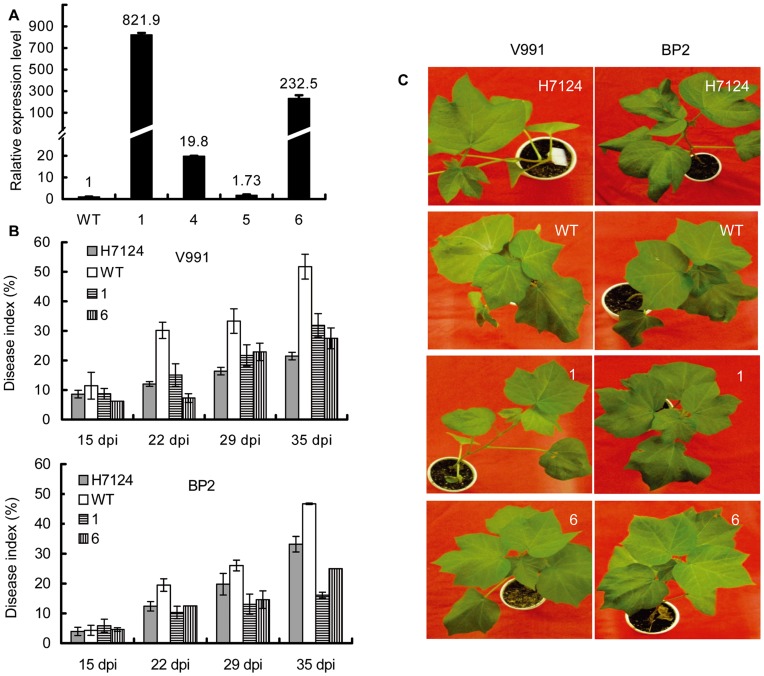
*Gbve1* confers Verticillium wilt resistance in transgenic upland cotton. (A). Relative expression level of *Gbve1* genes in T_1_ transgenic cotton lines. (B). The disease indices of T1 transgenic cottons with infection by *V. dahliae* defoliating isolate V991 (upper panel) and non-defoliating isolate BP2 (down panel). Results were presented as means±SE from three replications with at least 10 plants per replication. (C). The phenotypes of transgenic cotton lines with infection by *V. dahliae* isolates.

The same vector carrying *Gbve1* was also transformed into the genome of *G. hirsutum* var. WC by *Agrobacterium*-mediated transformation of embryogenic calli. Twelve independent T_0_ transformed plants were generated under kanamycin selection and transplanted into pots for greenhouse maturation (Figure S3). All of these putative transgenic cotton plants had similar phenotypes to the non-transformed negative controls with respect to growth, leaf shape, and flowering (Figure S3). The integration of the *Gbve1* construction into the cotton genome was confirmed by PCR analysis and Southern blotting with the DIG-labeled *NPTII* gene as the probe (Figure S4). According to the Southern blot result, T_1_ plants of four transgenic lines (1, 4, 5 and 6) were subjected to further analysis. The expression of the *Gbve1* gene was measured by qRT-PCR. The transgenic line 1 yielded the highest expression of *Gbve1* at 822- fold than the wild type, while the transgenic line 6, 4 and 5 showed 233-, 20-, and 2-fold expression, respectively, compared to the WT control (Figure 6A). Two single-copy transgenic lines (1 and 6) with high expression of *GbVe1* gene, were further applied to analyze the resistance to *V. dahliae* isolate V991 and BP2. Disease evaluation showed that these two transgenic lines displayed disease indices significantly lower than those of the WT throughout the developmental stages. The resistance of the line 1 and 6 to *V. dahliae* was both comparable with that in *G. barbadense* H7124 (Figure 6B, 6C). The disease index of transgenic lines to defoliating isolate V991 was slightly higher than that of H7124 (Figure 6B). However, the line1 and 6 demonstrated better resistances to non-defoliating isolate BP2 than that of H7124 (Figure 6B).

## Discussion

Many island cotton cultivars are resistant or near immune to Verticillium wilt [3], while most upland cotton cultivars are sensitive to this notorious disease [43]. A single dominant *Ve* locus that encodes *Ve1* and *Ve2* genes confers resistance to race 1 *Verticillium* strains and has been widely used in tomato breeding [16], [44]. However, no race-specific resistant gene has been identified in cotton. Here, we cloned a *Ve1*-like gene, *GbVe1*, from island cotton by screening the BAC library and presented several lines of evidence to support that the *GbVe1* gene might be an important component in protecting island cotton from Verticillium wilt. Importantly, we found that the levels of resistance to *V. dahliae* isolates of two testing transgenic lines were equivalent to that of island cotton.

Several *Ve1*/*Ve2* like genes have been cloned from *S. licopersicoides* [40], *S. torvum* [41], *G. hirsutum* [26], and *G. barbadense* [7]. Those proteins share high sequence similarity with each other and contain almost identical domains (Figure 1). Gene silencing of the *GhVe1* gene by VIGS in cotton seedlings increased its sensitivity to *V. dahlia* [26], which is consistent with the results of the *GbVe1* gene used in this study (Figure 4). The cotton genome might encode several *Ve1*/*Ve2* homologues that share high sequence similarity with each other. Thus, functional analysis by VIGS suggests that this gene family is necessary for resistance against Verticillium wilt. Since *Arabidopsis* is also a host for *V. dahliae*, it serves a good model plant to analyze *Ve*-relevant genes. Transgenic *Arabidopsis* lines with *Ve1* gene [18] and *GbVe* gene [7] had improved resistance levels to non-defoliating isolates of *V. dahliae*. Here, we showed that *GbVe1*-mediated strong resistance to both defoliating and non-defoliating isolates of *V. dahliae* in *Arabidopsis* and upland cotton. We also cloned *GbVe1* alleles from both susceptible and tolerant upland cotton. Transcriptional analysis showed that the response times, and induction levels, were dramatically different between incompatible and compatible interactions. From these results, we inferred that *GbVe1* conferred high resistance to the defoliating and non-defoliating isolates of *V. dahliae*.

*Ve1*-mediated resistance in tomato is activated by an avirulence protein (Ave1) encoded by race 1 *V. dahliae* strains. A homolog of the *Ave1* gene was also found in the bacterial plant pathogen *Xanthomonas axonopodis* and in several other plant pathogenic fungi [23], but not in the tested *V. dahliae* strains that are responsible for cotton Verticillium wilt [45]. *Gbve1*-mediated resistance in *Arabidopsis* and cotton might be triggered by other unknown pathogen-associated molecular patterns (PAMPS) or effectors that are encoded by the tested *V. dahliae* strains. Our results also indicate that the expression of the *Gbve1* gene is activated by SA, ETH, and JA treatment (Figure 2). Those signaling molecules play important roles in basal plant defense responses as well as in gene-for-gene-mediated defense [46], and are also crucial in Verticillium wilt resistance [47]. We suggest that Verticillium wilt resistance may be part of a complex, multi-hormone signal network, and the expression of the *Gbve1* gene in cotton might be partially through signaling molecules after infection by *V. dahliae*. Regardless of what are the resistance mechanisms and downstream signaling components for the GbVe1 protein, we have demonstrated here that the GbVe1 from Island cotton confers the resistance to *V. dahliae* in cotton and *Arabidopsis*. Our results support the recent findings that a *GbVe1* allele, *GbVe*, from *G. barbadense* also contributes resistance to *V. dahliae* when it is over-expressed in *Arabidopsis* [7].

Currently, the best way to prevent *Verticillium* diseases is the use of resistant cultivars. As noted above, highly resistant cultivars are usually absent in upland cottons, and different strategies to develop transgenic cotton with increased levels of *Verticillium* resistance have been tested. However, the resistance level of the transgenic plant is usually limited and not sufficient to generate durable resistance. For example, *AtNPR1*-transgenic cotton seedlings increase resistance only to the weak pathogens [8], and the resistance levels of transgenic seedlings were evaluated only in a naturally infested Verticillium wilt nursery, with information on pathogens not being included [7], [10], [11], [15]. Here, we measured the transgenic cotton lines in a greenhouse and found that the *Gbve1* gene dramatically increased the resistance level to both defoliating and non-defoliating isolates of *V. dahliae*. We suggest that both *Gbve1* and *Gbve* genes might be useful in the breeding of cotton varieties resistant to Verticillium wilt.

## Supporting Information

Figure S1 **Primary structures of the five Ve related proteins.**
(TIF)

Figure S2 **Phenotypes of gene silenced lines of** ***Gossypium barbadense*** **.** WT: without agroinfection treatment, CLCrV-A-vdr2+ CLCrV-B: agroinfection with vector CLCrV-A-vdr2 and CLCrV-B, CLCrV-A+ CLCrV-B: agroinfection with empty vector CLCrV-A and CLCrV-B
(TIF)

Figure S3 **The transformation of cotton embryogenic calli with** ***Agrobacterium*** **.** (A). Embryogenic calli for *Agrobacterium* transformation. (B). Kanamycin resistant embryogenic calli after selective culture. (C). Embryogenesis of kanamycin resistant embryogenic calli. (D). Regenerated plantlets in culture. (E). Regenerated plantlets transferred in pots. (F). Transgenic plants in greenhouse.
(TIF)

Figure S4 **PCR analysis and Southern blot of transgenic cotton plants.** (A). PCR analysis of independent T_0_ transgenic cotton plants with the region of CaMV35S and the *Gbve1* gene (1013 bp). M: DL2000 marker; P: plasmid DNA; H: H_2_O; 1–14: independent regenerated cotton plants. (B). Southern blot of independent T_0_ transgenic cotton plants. M: lambda-*Eco*T14 I digest DNA marker; WT: untransformed plant control; NT: non-transgenic regenerated plant without target genes; line 1–6: putative transgenic cotton plants confirmed by PCR analysis. Line 1 and 6 showed a single-copy integration, line 4 had at least six bands, line 5 may pose two hybridization signals, while line 2 and 3 may be *NPTII* negative lines.
(TIF)

Table S1 **The characterized domains in the promoter of** ***Gbve1*** **gene.**
(DOC)

Table S2 **The used primers in this study.**
(DOC)
