# Supplementary figures and images for: Island Cotton Gbve1 Gene Encoding A Receptor-Like Protein Confers Resistance to Both Defoliating and Non-Defoliating Isolates of Verticillium dahliae
Source: PLoS One. 2012 Dec 10;7(12):e51091. doi: 10.1371/journal.pone.0051091 (PMC3519487; doi:10.1371/journal.pone.0051091)

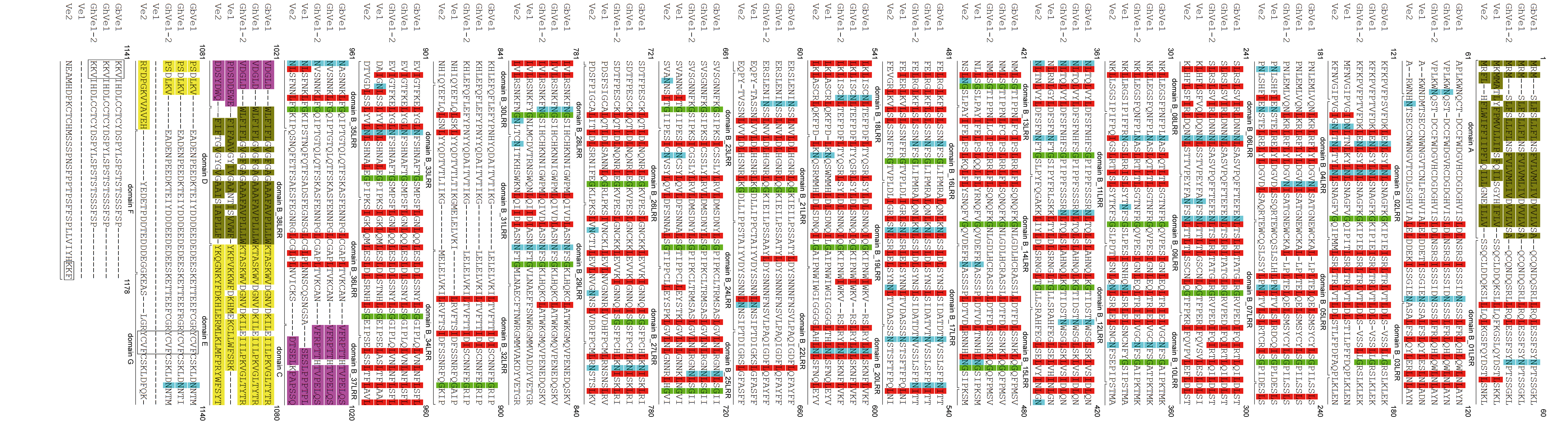

Supplement: Figure S1 — Primary structures of the five Ve related proteins. (TIF) [file pone.0051091.s001.tif]

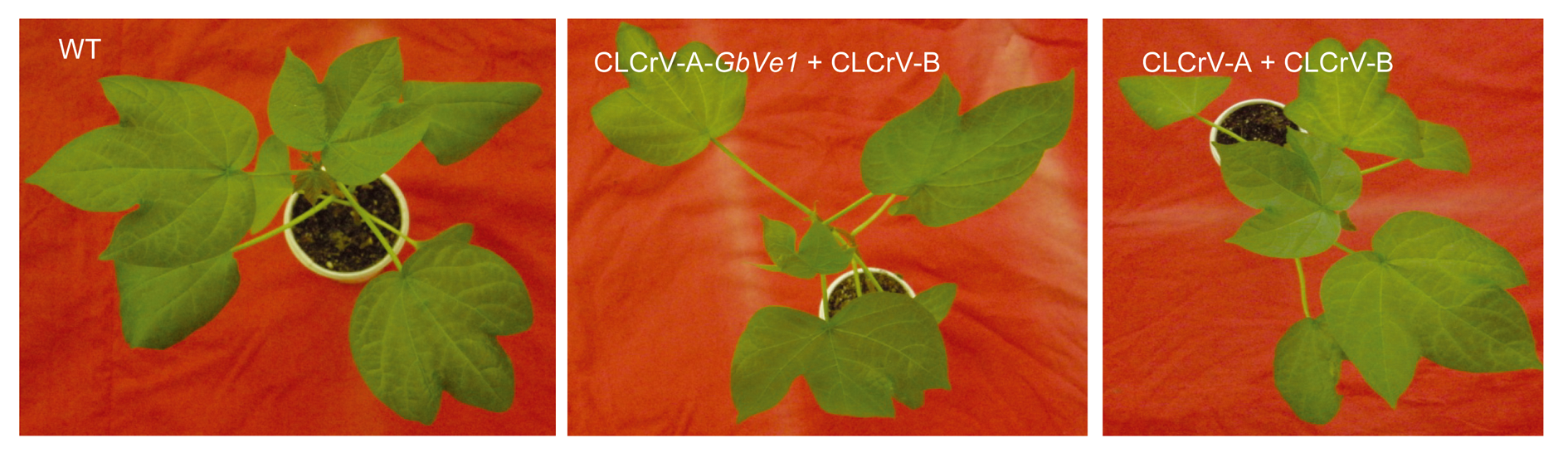

Supplement: Figure S2 — Phenotypes of gene silenced lines of Gossypium barbadense . WT: without agroinfection treatment, CLCrV-A-vdr2+ CLCrV-B: agroinfection with vector CLCrV-A-vdr2 and CLCrV-B, CLCrV-A+ CLCrV-B: agroinfection with empty vector CLCrV-A and CLCrV-B (TIF) [file pone.0051091.s002.tif]

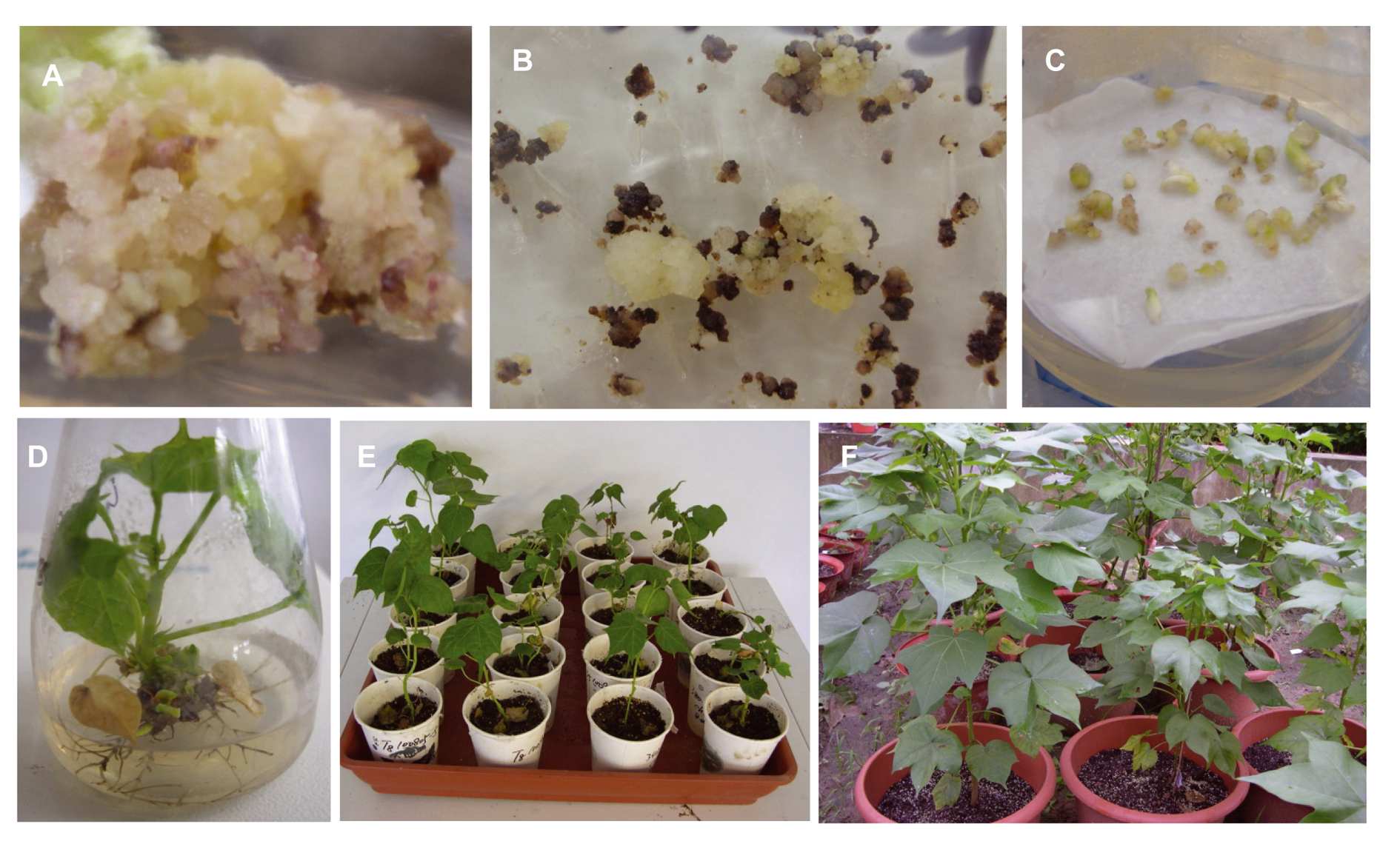

Supplement: Figure S3 — The transformation of cotton embryogenic calli with Agrobacterium . (A). Embryogenic calli for Agrobacterium transformation. (B). Kanamycin resistant embryogenic calli after selective culture. (C). Embryogenesis of kanamycin resistant embryogenic calli. (D). Regenerated plantlets in culture. (E). Regenerated plantlets transferred in pots. (F). Transgenic plants in greenhouse. (TIF) [file pone.0051091.s003.tif]

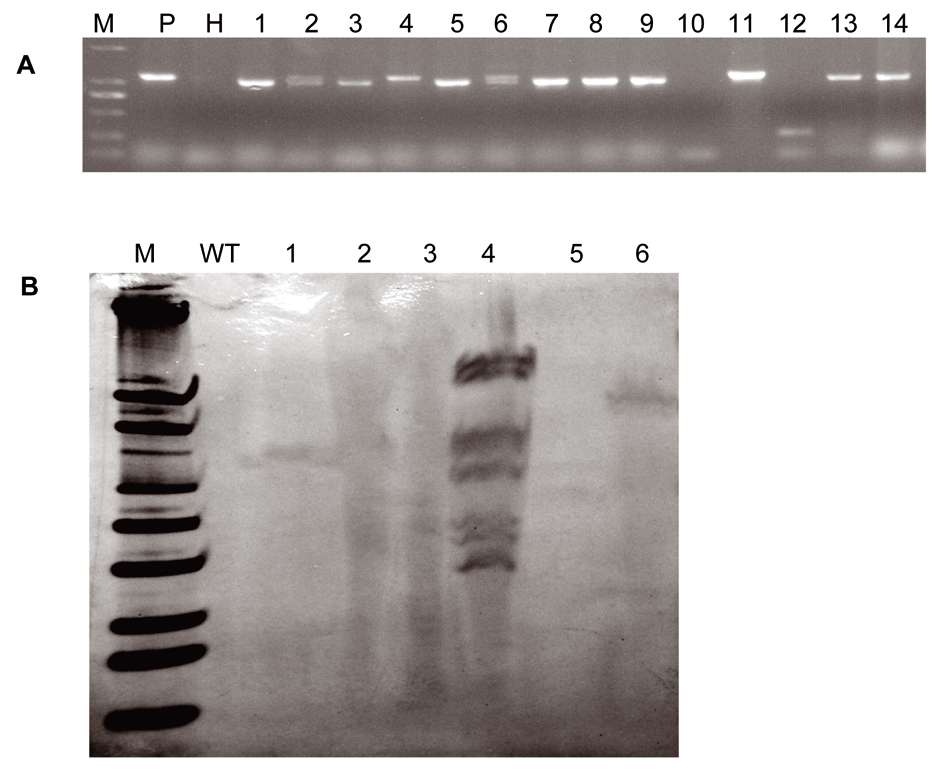

Supplement: Figure S4 — PCR analysis and Southern blot of transgenic cotton plants. (A). PCR analysis of independent T0 transgenic cotton plants with the region of CaMV35S and the Gbve1 gene (1013 bp). M: DL2000 marker; P: plasmid DNA; H: H2O; 1–14: independent regenerated cotton plants. (B). Southern blot of independent T0 transgenic cotton plants. M: lambda-EcoT14 I digest DNA marker; WT: untransformed plant control; NT: non-transgenic regenerated plant without target genes; line 1–6: putative transgenic cotton plants confirmed by PCR analysis. Line 1 and 6 showed a single-copy integration, line 4 had at least six bands, line 5 may pose two hybridization signals, while line 2 and 3 may be NPTII negative lines. (TIF) [file pone.0051091.s004.tif]
