# Supplementary material for: Island Cotton Gbve1 Gene Encoding A Receptor-Like Protein Confers Resistance to Both Defoliating and Non-Defoliating Isolates of Verticillium dahliae
Source: PLoS One. 2012 Dec 10;7(12):e51091. doi: 10.1371/journal.pone.0051091 (PMC3519487; doi:10.1371/journal.pone.0051091)
Supplement: Table S1 — The characterized domains in the promoter of Gbve1 gene. (DOC) [file pone.0051091.s005.doc]

Table S1 **Some domains in the promoter of *Gbve1* gene**

| SEQUENCE | LOCATION | NAME AND FUNCTION | REFERENCE |
| --- | --- | --- | --- |
| TGACG | -234 | ASF1MOTIFCAMV, auxin and/or salicylic acid activation | Despres C, Chubak C, Rochon A, et al. The Arabidopsis NPR1 disease resistance protein is a novel cofactor that confers redox regulation of DNA binding activity to the basic domain/leucine zipper transcription factor TGA1. Plant Cell 15: 2181-2191 (2003) |
| TGTCA | -292 | BIHD1OS, binding site of OsBIHD1 and activation of disease resistant genes | Luo H, Song F, Goodman RM, et al. Up-regulation of OsBIHD1, a rice gene encoding BELL homeodomain transcriptional factor, in disease resistance responses. Plant Biol. 7: 459-468 (2005). |
| AWTTCAAA | -1134 | ERELEE4, ethylene-induced activation | Itzhaki H, Maxson JM, Woodson WR. An ethylene-responsive enhancer element is involved in the senescence-related expression of the carnation glutathione-S-transferase (GSTI) gene. Proc Natl Acad Sci USA 91:8925-8929 (1994) |
| AACGTGT | -296 | QARBNEXTA, wounding and tensile stress responsive element | Elliott KA , Shirsat AH. Promoter regions of the extA extensin gene from Brassica napus control activation in response to wounding and tensile stress. Plant Mol Biol 37:675-687 (1998) |
| TTGAC | -235 | WBOXATNPR1, salicylic acid (SA) responsive element | Yu D, Chen C, Chen Z. Evidence for an important role of WRKY DNA binding proteins in the regulation of NPR1 gene expression. Plant Cell 13: 1527-1540 (2001) |
| CTGACY | -1470 | WBOXNTCHN48, Elicitor-respsonsive transcription element | Yamamoto S, Nakano T, Suzuki K, Shinshi H. Elicitor-induced activation of transcription via W box-related cis-acting elements from a basic chitinase gene by WRKY transcription factors in tobacco. Biochim Biophys Acta. 1679:279-287(2004). |
| TGACY | -784, -625 | WBOXNTERF3, wound-respsonsive transcription element | Nishiuchi T, Shinshi H, Suzuki K. Rapid and transient activation of transcription of the ERF3 gene by wounding in tobacco leaves: Possible involvement of NtWRKYs and autorepression. J Biol Chem. 279: 55355-55361 (2004) |
