# Supplementary material for: Island Cotton Gbve1 Gene Encoding A Receptor-Like Protein Confers Resistance to Both Defoliating and Non-Defoliating Isolates of Verticillium dahliae
Source: PLoS One. 2012 Dec 10;7(12):e51091. doi: 10.1371/journal.pone.0051091 (PMC3519487; doi:10.1371/journal.pone.0051091)
Supplement: Table S2 — The used primers in this study. (DOC) [file pone.0051091.s006.doc]

Table S2 **Primers in this study**

| **No.** | **Primer** | **Applications** | **Sequence(from 5' to 3')** |
| --- | --- | --- | --- |
| 1 | EST- F | Prepare probe from cotton EST for BAC screening | TTCTGGTCCAATACCATCATTCT |
| 2 | EST -R |  | CTTAGATTCAGTACTCCAAGAGA |
| 3 | Vdr2-cF | Cloning and insertion of *Gbve1* gene into pGEM-T Eeasy vector | TGACCCGGGATTGATACTAATGAGGATGTCACTC |
| 4 | Vdr2-cR |  | TCAGAGCTCTTTCATCACCCCTTTCCATGGT |
| 5 | AP1 | Cloning promoter of *Gbve1* gene with genome walking (Round I ) | GTAATACGACTCACTATAGGGC |
| 6 | GSP1 |  | TGGTAGGCAAAAGAAAGAACCGTCA |
| 7 | AP2 | Cloning promoter of *Gbve1* gene with genome walking (Round II ) | ACTATAGGGCACGCGTGGTC |
| 8 | GSP2 |  | AGAATCGAGACTTGCGGCAGAAACA |
| 9 | Vdr2-qF | qRT-PCR for *Gbve1* gene | TTTCGACCTACAACACATACAGTG |
| 10 | Vdr2-qR |  | CTTCGTCTTCTTCATCATCGTCAT |
| 11 | UBQ14-qF | Internal control of qRT-PCR for UBQ14 gene | CAACGCTCCATCTTGTCCTT |
| 12 | UBQ14-qR |  | TGATCGTCTTTCCCGTAAGC |
| 13 | Gbve1-*Sma*IF | Cloning and insertion of *Gbve1* gene into pCAMBIA2301 binary vector | TGACCCGGGATTGATACTAATGAGGATGTCACTC |
| 14 | Gbve1-*Sac*IR |  | TCAGAGCTCTTTCATCACCCCTTTCCATGGT |
| 15 | Pro-F | Cloning and insertion of *Gbve1* promoter into pBI101.1 binary vector | tgaggatccGATAGCAATGGCACTAGGTTGAG |
| 16 | Pro-R |  | tgagtcgacAGGGTGTTCACTGTCTTGTGGTT |
| 17 | PR1-qF | qRT-PCR for *PR1* gene in transgenic *Arabidopsis* | TCAGTGAGACTCGGATGTGC |
| 18 | PR1-qR |  | CGTTCACATAATTCCCACGA |
| 19 | PR5-qF | qRT-PCR for *PR5* gene in transgenic *Arabidopsis* | CTCTTCCTCGTGTTCATCACA |
| 20 | PR5-qR |  | TCAATTCAAATCCTCCATCG |
| 21 | EDS1-qF | qRT-PCR for *EDS1* gene in transgenic *Arabidopsis* | CGAAGGGGACATAGATTGGA |
| 22 | EDS1-qR |  | ATGTACGGCCCTGTGTCTTC |
| 23 | GST1-qF | qRT-PCR for *GST1* gene in transgenic *Arabidopsis* | AAAGAGCCTTTCATCCTTCG |
| 24 | GST1-qR |  | TGTCCTTGCCAGTTGAGAGA |
| 25 | TUB-qF | Internal control of qRT-PCR for *TUB* gene in transgenic *Arabidopsis* | GAGGGAGCCATTGACAACATCTT |
| 26 | TUB-qR |  | GCGAACAGTTCACAGCTATGTTCA |
| 27 | Vdr2-vF | Cloning and insertion a fragment of *Gbve1* gene into CLCrV vector for cotton VIGS | ACTAGTCGAGAAGCCAAACCTAGAGAT |
| 28 | Vdr2-vR |  | TTAATTAAGGTAAGTGCGAGAGTTCGAA |
| 29 | Clcrv-F | PCR check the replication of CLCrV in VIGS treated cotton plantlets | AAGCTTACCTGAACTTCCAAGTCTGGA |
| 30 | Clcrv-R |  | GCCTAATGGGTATAGAGCAAAATGGCA |
